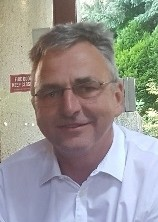# Marine Polysaccharides: Occurrence, Enzymatic Degradation and Utilization

**DOI:** 10.1002/cbic.202100078

**Published:** 2021-05-14

**Authors:** Marcus Bäumgen, Theresa Dutschei, Uwe T. Bornscheuer

**Affiliations:** ^1^ Department of Biotechnology & Enzyme Catalysis Institute of Biochemistry, University of Greifswald 17487 Greifswald Germany

**Keywords:** carrageenan, CAZymes, laminarin, marine polysaccharides, porphyran, ulvan

## Abstract

Macroalgae species are fast growing and their polysaccharides are already used as food ingredient due to their properties as hydrocolloids or they have potential high value bioactivity. The degradation of these valuable polysaccharides to access the sugar components has remained mostly unexplored so far. One reason is the high structural complexity of algal polysaccharides, but also the need for suitable enzyme cocktails to obtain oligo‐ and monosaccharides. Among them, there are several rare sugars with high value. Recently, considerable progress was made in the discovery of highly specific carbohydrate‐active enzymes able to decompose complex marine carbohydrates such as carrageenan, laminarin, agar, porphyran and ulvan. This minireview summarizes these achievements and highlights potential applications of the now accessible abundant renewable resource of marine polysaccharides.

## Graphical abstract

**Algae biomass is a source** for many valuable compounds for the pharmaceutical and chemical industry. Especially marine polysaccharides contain rare sugars of interest, but a major bottleneck is access to these sugars. In this mini‐review the common knowledge of marine CAZyme‐based deconstruction of these polysaccharides and the biotechnological potential of the thus accessible oligo‐ and monosugars are described.

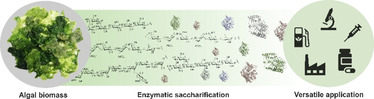

## 1 Introduction

**Scheme 1:**
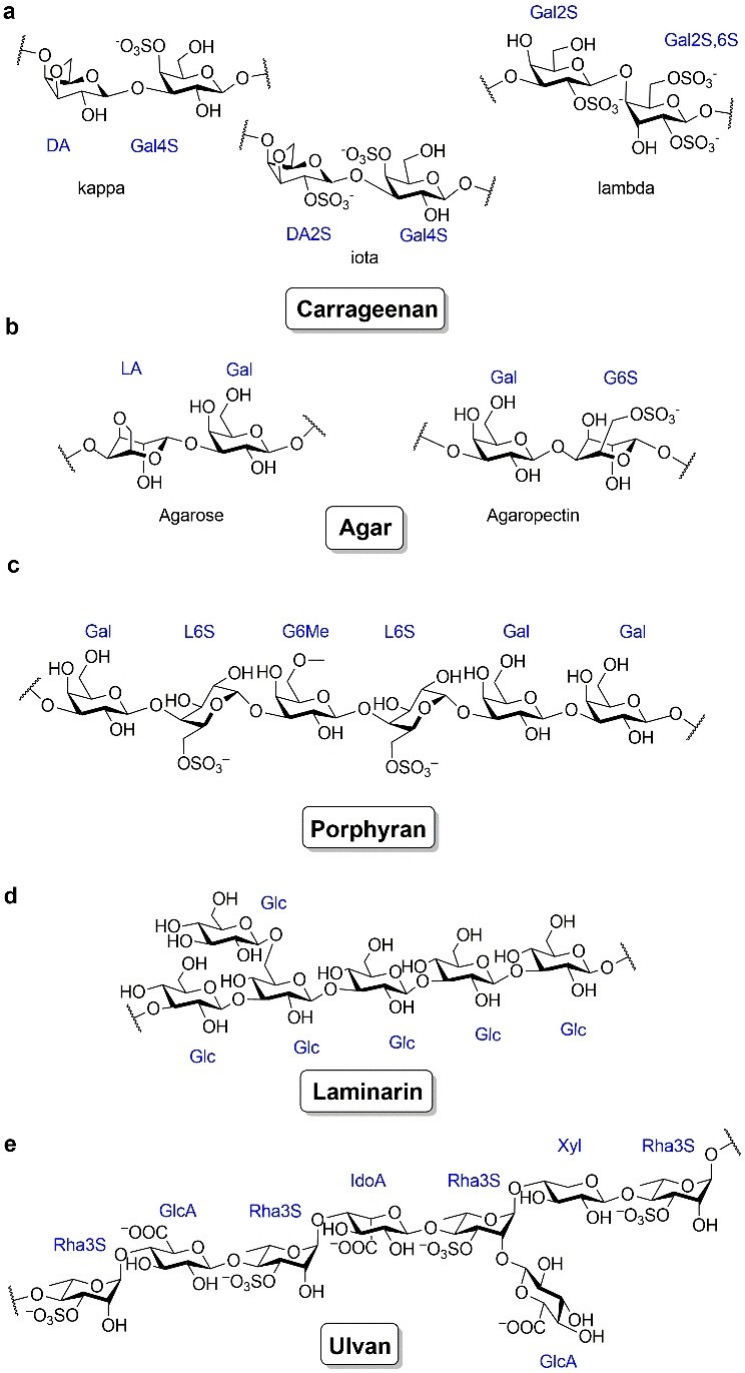
Structures of the marine polysaccharides carrageenan (a), agar (b), porphyran (c) laminarin (d) and ulvan (e). Carrageenan is composed of 3,6‐anhydro‐d‐galactose (DA) and d‐galactose (Gal). Agar divides in agarose and agaropectin which contains Gal and 3,6‐anhydro‐l‐galactose (LA). Laminarin contains d‐glucose. Porphyran is composed of d‐galactose and L‐galactose (L). Ulvan is composed of d‐glucuronic acid, l‐iduronic acid (IdoA), d‐xylose (Xyl) and l‐rhamnose (Rha). A number in combination with an ‘S’ attached to a sugar represents the position of sulfate groups. A number in combination with a ‘Me’ attached to a sugar represents the position of methyl groups.

The marine realm covers 70 % of the earth's surface making the oceans the largest ecosystem on earth,^[1]^ which may contain over 80 % of world's plant and animal species.^[2]^ In particular, the marine systems have great influence on the atmospheric CO_2_ concentration as the oceans contain the largest carbon pool in the carbon cycle.^[3]^ The increased level of atmospheric carbon dioxide, however, leads to a higher absorption rate by the world's oceans, resulting in a decreased pH‐value.^[4]^ Consequently, the carbonate concentration in the surface water is reduced, making the ocean acidification a disturbing effect for the aquatic carbonate chemistry, which is of great importance for marine calcifying organisms like molluscs, crustaceans, echinoderms, corals, large calcareous algae, foraminifera and some phytoplankton.^[5]^ Besides the increasing CO_2_ concentration on earth, the eutrophication of the oceans has a huge impact on the marine ecosystem. The increasing nutrient supply can cause an immense proliferation of algae, so called ‘algae blooms’ like the ‘Golden tides’, which are formed by the genus *Sargassum* in the Atlantic ocean or the ‘Green tides’, which are formed by the genus *Ulva* and occur worldwide.[^6^, ^7^] Beside the harmful environmental effects and high disposal cost of algal waste, the rising occurrence of algal biomass from these blooms also has a huge potential for biotechnological applications. One bottleneck for its use is access to the valuable chemical compounds within the algae, which has been described in recent reviews for the lipids and protein fractions.[^8^, ^9^, ^10^] For marine polysaccharides, Trincone provided an overview about carbohydrate‐active enzymes (CAZymes) involved in the degradation of macroalgal polysaccharides^[11]^ and Filote *et al*. covered aspects of potential biorefinery processes utilizing marine sugars.^[8]^ In the review by Ertesvåg, the enzymatic degradation pathway of alginate was illustrated^[12]^ which is complemented by a recent summary of the characteristics and applications of alginate lyases^[13]^ and new insights into fungal alginate lyases from *Paradendryphiella salina*.^[14]^ However, a detailed article dealing with the enzymatic degradation of other complex marine polysaccharides to access rare sugars is missing. This minireview therefore focuses on the current status of the microbial decomposition of the marine polysaccharides carrageenan, laminarin, agar, porphyran and ulvan (Scheme 1). We aim to provide an overview of the complexity of marine polysaccharides and the ubiquitous potential of this carbon source in biotechnological applications.

## 2 Diversity of Marine Carbohydrate Structures

Carbohydrates represent the largest proportion of marine biomass. They mainly occur in marine plants, macro‐ and microalgae[^15^, ^16^, ^17^] and can represent more than 50 % of the algal dry weight.[^18^, ^19^, ^20^] Many organisms use polysaccharides as intracellular energy storage compounds as well as structural cell wall components^[21]^ or secrete them as extracellular polymeric substances (EPS) with various functions.^[22]^ The polysaccharide composition varies substantially depending on the type of algae. Red algae mainly produce sulfated galactans, which are generally divided into agarans and carrageenans. While ulvan is the main polysaccharide in green algae, brown algae are known for the production of fucans.^[23]^ The polysaccharides of diatoms contain sulfated glucuronomannans and laminarin.[^24^, ^25^, ^26^, ^27^] The differences between terrestrial and marine carbohydrates originate in the variety of carbohydrate structures in their backbone as well as various modifications (Table 1). This is believed to be an adaption to the marine environment.[^26^, ^28^, ^29^] In comparison to freshwater and soil, the oceans contain a higher concentration of sulfate[^30^, ^31^] allowing for distinct sulfation patterns of the carbohydrates.[^26^, ^28^, ^29^] Due to the anionic properties of marine polysaccharides, especially through sulfation, algae presumably are resistant to desiccation,^[32]^ osmotic stress and heavy metal toxicity^[33]^ as well as more extreme temperature and pH values.^[34]^ The side group modifications and decorations of the carbohydrates further increase the algae's recalcitrance to degradation by enzymatic attack. This drives the adaptation of marine organism, especially bacteria, to develop specific enzymes which can remove these modifications from the carbohydrate backbone and then use common CAZymes to hydrolyse the glycosidic sugar bonds. An overview of the diversity of selected carbohydrates from marine algae and their monosaccharide composition is given in Table 1.

**Table 1:** Overview of marine algae carbohydrates and organisms of marine origin containing characterized CAZymes. The different marine polysaccharides are listed with their monosaccharide composition, methylation‐ and sulfation‐patterns. Furthermore, their main chain linkages and the occurrence of the corresponding polysaccharides in marine habitats are summarized. Marine organisms with characterized CAZymes for the degradation of the corresponding polysaccharide are also listed.

	Sugar composition^[a]^	−CH_3_^[b]^	−OSO_3_^−[b]^	Marine occurrence	Major CAZyme^[c]^	Marine polysaccharide degrader^[d]^
Agar^[e]^	β‐1,4‐d‐Galactose α‐1,3‐3,6‐Anhydro‐l‐galactose α‐1,3‐d‐Galactose	+	+	Red algae	GH16, GH117, GH50, α‐Agarase EC. 3.2.1.158 β‐Agarase EC 3.2.1.81	*Zobellia galactanivorans*,^[35]^*Saccharophagus degradans*,^[36]^*Alterococcus agarolyticus*,^[37]^*Flammeovirga* sp. SJP92,^[38]^
Alginate	β‐1,4‐d‐Mannuronic acid α‐1,4‐l‐Guluronic acid	+	−	Brown algae	PL7 Mannuronate lyase EC 4.2.2.3 Guluronate lyase EC 4.2.2.11	*Sphingomonas* sp. MJ‐3,^[39]^ *Microbulbifer* sp. ALW1,^[40]^ *Flavobacterium* sp. UMI‐01^[41]^
Carrageenan	β‐1,4‐d‐Galactose α‐1,3‐3,6‐Anhydro‐d‐galactose	+	+	Red algae	GH16 Carrageenase EC 3.2.1.83	*Pseudoalteromonas atlantica*,^[42]^*Zobellia galactanivorans*,^[43]^*Pseudoalteromonas carrageenovora* 9T,[^44^, ^45^, ^46^]
Cellulose	β‐1,4‐d‐Glucose β‐1,6‐d‐Glucose	−	−	Green and Brown algae	GH48, GH17, GH16, GH9 *Endo*‐glucanase EC 3.2.1.6 *Exo*‐glucanase EC 3.2.1.74	*Glaciecola* sp. 4H‐3‐7+YE‐5^[47]^, *Actinoalloteichus* sp. MHA15^[48]^, *Exiquobacterium* sp. Alg‐S5^[49]^
Fucoidan	α‐1,3‐l‐Fucose, α‐1,2‐l‐Fucose α‐1,2‐d‐Glucuronic acid	−	+	Brown algae	GH29, GH107, GH168 α‐l‐Fucosidase EC 3.2.1.51 α‐1,3–1,4‐l‐Fucosidase EC 3.2.1.111 *Endo*‐Fucoidanase EC 3.2.1.212	*Luteolibacter algae* H18,^[50]^ *Wenyingzhuangia fucanilytica*,^[51]^ *Lamellidens corrianus*,^[52]^ *Vibrio* sp. EJY3^[53]^
Laminarin	β‐1,3‐d‐Glucose β‐1,6‐d‐Glucose	−	−	Brown algae and diatoms	GH5 β‐1,3‐Glucanase EC 3.2.1.6	*Formosa agariphila* GH17A,^[54]^ *Formosa* sp. nov strain Hel1_33_131,^[54]^ *Pseudocardium sachalinensis*,^[54]^ *Vibrio campbellii*,^[55]^
Mannan	β‐1,4‐d‐Mannose α‐1,4‐d‐Mannose	−	−	Red and Green algae	GH5 β‐Mannanase EC 3.2.1.78	*Streptomyces* sp. Alg‐S25^[56]^
Pectin	α‐1,4‐d‐Galacturonic acid, α‐1,6‐d‐Galactose, β‐1,4‐d‐Xylose α‐1,5‐l‐Arabinose α‐1,2‐d‐Apiose α‐1,2‐l‐Rhamnose	+	−	Green algae and diatoms	PL1, PL2, PL3 Pectin lyase EC 4.2.2.10	*Pseudoalteromonas* sp. PS47,^[57]^ *Pseudoalteromonas haloplanktis* ANT/505^[58]^
Porphyran	β‐1,4‐d‐Galactose α‐1,3‐l‐Galactose	+	+	Red algae	GH16, GH86 β‐Porphyranase EC 3.2.1.178	*Z. galactanivorans*.^[59]^*Bacteroides plebeius*^[60]^
Ulvan	β‐1,4‐d‐Xylose α‐1,4‐l‐Iduronic acid, β‐1,4‐d‐Glucuronic acid, α‐1,4‐l‐Rhamnose	+	+	Green algae	PL24, PL25, PL28 Ulvan lyase EC 4.2.2.–	*Formosa agariphila*[^61^, ^62^]
Xylan	β‐1,4‐d‐Xylose^[f]^ β‐1,3‐d‐Xylose^[f]^	+	+	Red and Green algae	GH10, GH11, GH30 *Endo*‐1,4‐β Xylanase EC 3.2.1.8	*Paraglaciecola mesophile* KMM24*1*,^[63]^ *Vibrio* sp. XY‐214,^[64]^ *Alcaligenes* sp. XY‐234,^[65]^ *Glaciecola* sp. 4H‐3‐7+YE‐5,^[47]^ *Psychrobacter* sp. Strain 2–7,^[66]^

[a] The most prominent monosaccharides are listed. [b] Methylation (−CH_3_) or sulfatation (−OSO_3_^−^) patterns of the polysaccharides are indicated. The potential occurrence of these monosaccharide decorations is marked with + or in their absence with −. [c] CAZyme families only represent the enzyme for initial depolymerisation of the polysaccharide. [d] Characterized CAZymes from marine organism refer mostly to examples published between 2016–2020.^[11]^ [e] Agar is composed of agarose and agaropectin. [f] Red algae xylan consists of mixed linked type β‐1,4‐d‐Xylose and β‐1,3‐d‐Xylose while green algae xylan contains mostly β‐1,3‐d‐Xylose.^[67]^

## 3 Enzymatic Degradation of Marine Polysaccharides

The ability to compose and decompose polysaccharides is crucial for the global carbon cycle. To use them as energy source, heterotrophic organisms require a suitable set of CAZymes in order to degrade them to monosaccharides, which can be further converted through the central sugar metabolism. Marine Bacteroidetes are specialized to use complex algal polysaccharides of different origins as nutrient and therefore have developed surprisingly complex and dedicated enzyme toolboxes. This is also reflected by the observation that recurrent patterns of dominant bacterial groups outgrow during phytoplankton blooms in the North Sea.^[68]^ Gene clusters encoding a set of enzymes and further proteins (i. e. for sugar transport) required to decompose algal polysaccharides are organized in Bacteroidetes in so‐called polysaccharide utilization loci (PULs). These encode a broad variety of CAZymes to decompose the complex polysaccharides.[^62^, ^69^] Without detailed knowledge on relevant enzyme functions, the guided degradation of marine polysaccharides *in vitro* is rather difficult.

The CAZy database (www.CAZy.org)[^70^, ^71^] lists CAZymes grouped by their enzyme class and genetic relationship. This presently includes 163 classes of glycoside hydrolases (GHs), 111 classes of glycosyl transferases (GTs), 40 classes of polysaccharide lyases (PLs), 18 classes of carbohydrate esterases (CEs) and 16 classes of enzymes with auxiliary activity (AAs). For the depolymerization of carbohydrates many different enzyme functions are necessary. There are *endo*‐active CAZymes, which cleave within the polysaccharide chain and *exo*‐enzymes, which remove saccharide fragments from the ends. Glycoside hydrolases are the most diverse family of CAZymes. They catalyse the hydrolysis of glycosidic bonds.^[72]^ In polysaccharides that contain uronic acid residues, like alginate or ulvan, polysaccharide lyases catalyse the non‐hydrolytic cleavage of the chain at an uronic acid residue via a β‐elimination mechanism.^[73]^ Several side groups increase the resistance against backbone‐cleaving enzymes. Besides further GHs that cleave off various monosaccharide side chains, other enzymes are required for the deprotection of the polysaccharide backbone. Polysaccharide sulfatases remove sulfate ester groups,^[26]^ while carbohydrate esterases catalyse the cleavage of *O*‐ and *N*‐acetyl groups from carbohydrates.^[74]^ In contrast to the CEs, the sulfatases are not implemented in the CAZy database but are listed in the SulfAtlas database instead.^[75]^ The class of ‘auxiliary activities‘ includes redox enzymes that act in conjunction with other CAZymes.^[76]^ This includes lytic polysaccharide monooxygenases (LPMOs) and enzymes known to be involved in lignin degradation. Another example for enzymes with auxiliary activities are the recently discovered cytochrome P450 monooxygenases from the marine bacteria *Formosa agariphila* and *Zobellia galactanivorans*. It was shown that they specifically catalyse the demethylation of 6‐*O*‐methyl‐d‐galactose present in the algal polysaccharides agarose and porphyran. Only after enzymatic hydroxylation and a subsequent decomposition step – yielding the free hydroxyl group of d‐galactose and formaldehyde – further degradation can occur.^[77]^ In the following, these complex pathways are highlighted for selected algal carbohydrates.

### 3.1 Carrageenan

Besides agars, carrageenans are the main cell wall polysaccharides of red macroalgae.^[78]^ Their structure is very complex and depend on the algal species. In general, they consist of sulfate esters of α‐1,3‐linked 3,6‐anhydro‐d‐galactose and β‐1,4‐linked d‐galactose (Table 1).^[11]^ The most prominent carrageenans for commercial applications are κ‐, ι‐ and λ‐carrageenan (Scheme 1).[^11^, ^32^] The decomposition of such complex polysaccharides to the monomeric level requires many different enzyme functions.

**Figure 1:**
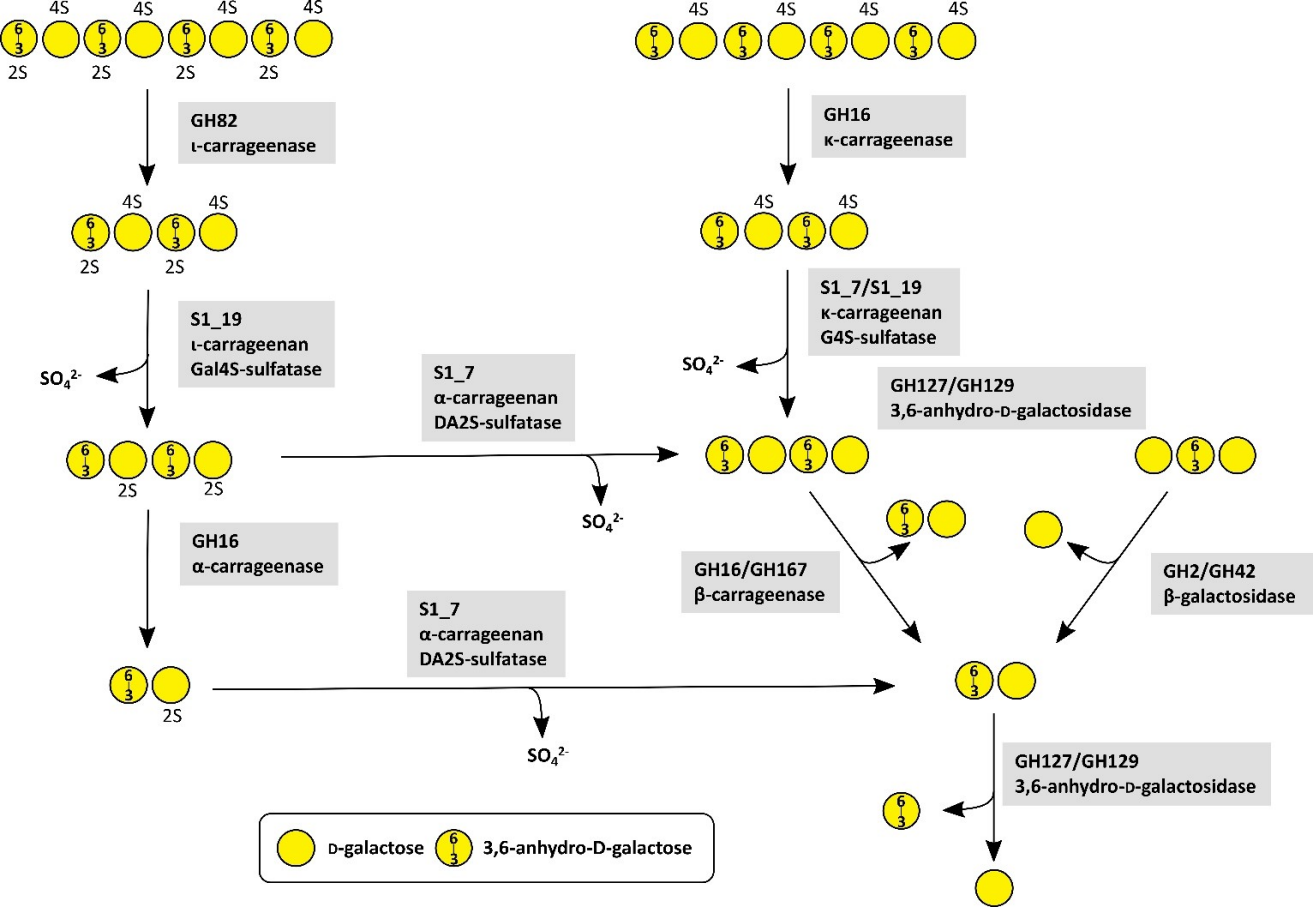
Metabolic carrageenan degradation pathway by CAZymes based on current knowledge.[^44^, ^45^, ^46^, ^78^] The oligosaccharides on the top represent a section of a larger polysaccharide chain. A number in combination with an ‘S’ attached to a sugar represents the position of sulfate groups.

The main carrageenan degrading enzymes are κ‐carrageenases (EC 3.2.1.83), ι‐carrageenases (3.2.1.157) and λ‐carrageenases (3.2.1.162). They cleave the β‐1,4‐linkages of polymeric carrageenans under the production of oligomeric neocarrabiose.^[79]^ The first ι‐carrageenase‐activity was reported for enzymes from *Alteromonas fortis* and *Z. galactanivorans* leading to the generations of GH family 82 which differs from κ‐carrageenases.^[79]^ These enzyme groups and their mode of action were reviewed a few years ago.^[80]^ As carrageenans are highly sulfated polysaccharides, the removal of sulfate groups is required for achieving complete decomposition (Figure 1). The first carrageenan sulfatase from *Pseudoalteromonas carrageenovora* was a 4‐*O*‐κ‐carrabiose sulfatase.^[81]^ The synergistic degradation of sulfated carrageenans by GHs and sulfatases was demonstrated for *Z. galactanivorans*.^[78]^ Here, ι‐ and κ‐carrageenan required a desulfation of the C4 sulfate group of d‐galactose by two specialized sulfatases resulting in α‐ or β‐carrageenan. A third sulfatase converts α‐carrageenan into desulfated β‐carrageenan by removing the C2 sulfate group from anhydro‐galactose. Without these desulfations further degradation steps by GHs were blocked.^[78]^ In detail, the first step of ι‐carrageenan degradation is the cleavage of the polysaccharide chain into smaller oligosaccharides by ι‐carragenases of family GH82. In κ‐carrageenan this first step is carried out by a κ‐carrageenase of family GH16. Oligomeric ι‐carrageenan requires a desulfation of the d‐galactose residues by an ι‐carrageenan G4S‐sulfatase from family S1_19 resulting in oligomeric α‐carrageenan. The same steps occur in κ‐carrageenan. Here, a κ‐carrageenan G4S‐sulfatase hydrolyses the sulfate ester at d‐galactose residues, leading to unsulfated β‐carrageenan. To convert α‐carrageenan into the unsulfated β‐carrageenan a desulfation of the remaining 3,6‐anhydro‐d‐galactose residues by an α‐carrageenan DA2S‐sulfatase from family S1_17 is required. Finally, the unsulfated β‐carrageenan can be successively degraded from the non‐reducing end by 3,6‐anhydro‐d‐galactosidases from family GH127 or GH129 proteins and β‐galactosidases from family GH2 (Figure 1).^[78]^ Recently the carrageenan decomposition was investigated in several *Pseudoalteromonas* species.^[82]^ Two GHs from family GH16 with high identity to a previously described GH16 family κ‐carrageenase from *P. carrageenovora* 9T[^44^, ^45^, ^46^] and a β‐carrageenan‐specific *endo*‐hydrolase from *Paraglaciecola hydrolytica* SS66T^[83]^ were able to degrade κ‐carrageenan into even numbered κ‐neocarrageenan oligosaccharides. The synergistic activity between previously described S1_19 sulfatase^[84]^ and these GH16 enzymes were revealed to resemble the previous results for *Z. galactanivorans*.^[78]^ A prior desulfation of κ‐carrageenan or ι‐carrageenan by the S1_19 sulfatase allowed depolymerisation by a third GH16 enzyme indicating an α‐ or β‐carrageenases activity. A second S1_19 sulfatase was revealed to be an *exo*‐G4S κ‐carrageenan sulfatase being inactive on ι‐carrageenan.^[82]^ A β‐neocarrabiose releasing *exo*‐carrageenase of family GH167 was shown to degrade short κ‐carrageenan oligosaccharides after treatment with κ‐carrageenan‐active sulfatase. This enzyme showed 63 % identity^[82]^ with a formerly described carrageenan‐active enzyme from *P. hydrolytica*.^[83]^

### 3.2 Porphyran

Agars are galactans from red algae containing α‐1,3‐linked l‐galactose and β‐1,4‐linked d‐galactose. l‐galactose is replaced by 3,6‐anhydro‐l‐galactose in agarose and by l‐galactose‐6‐sulfate in porphyran (Scheme 1, Table 1).[^32^, ^85^] Porphyran is especially abundant in algae of the genus *Porphyra*.

The degradation of agars in general was reviewed before,^[86]^ as were the biochemical characterizations of agarose‐degrading pathways.[^87^, ^88^]

**Figure 2:**
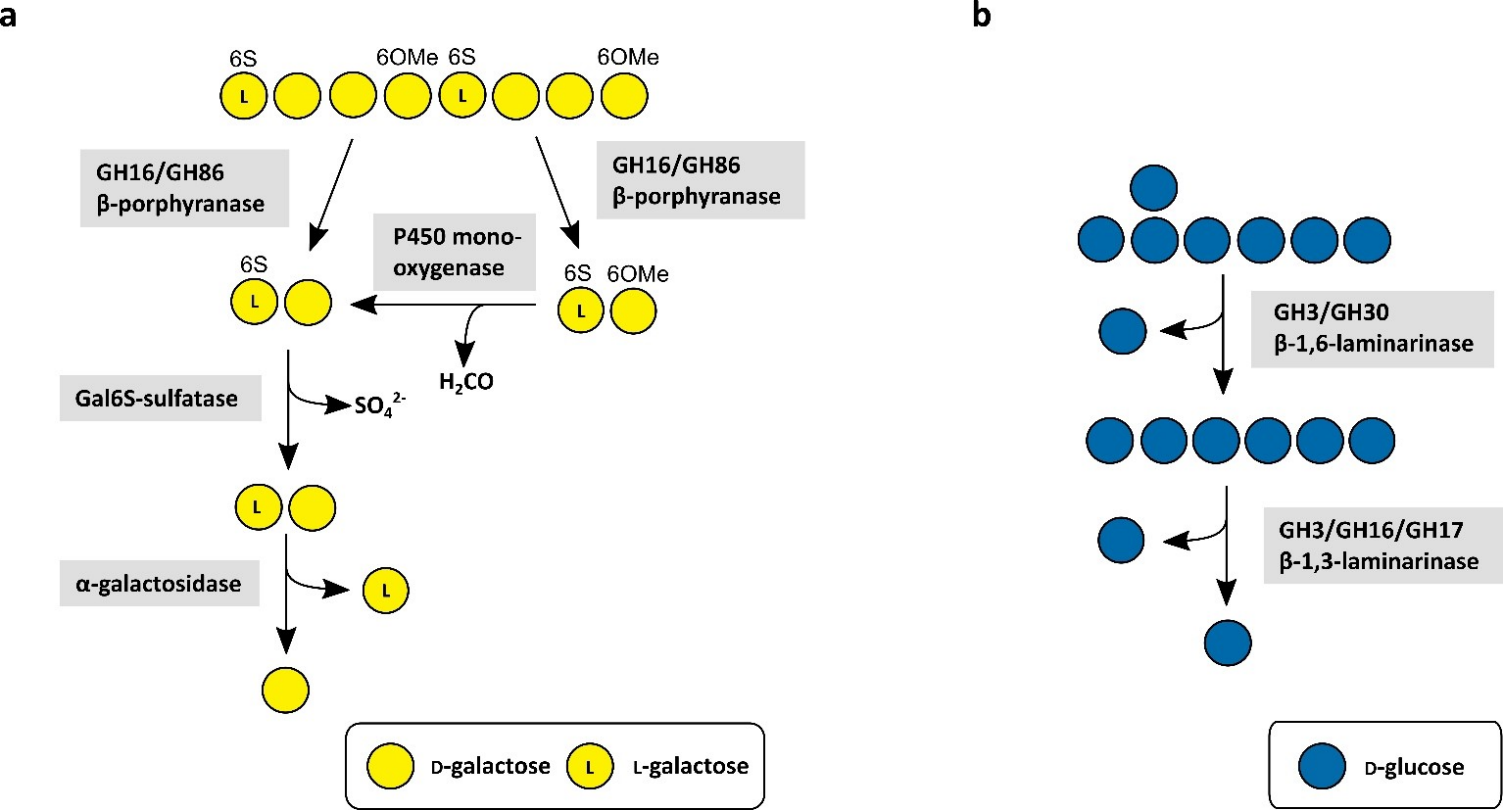
Metabolic porphyran (**a**) and laminarin (**b**) degradation pathways by CAZymes based on current knowledge.[^54^, ^55^] The oligosaccharides on the top represent a section of a larger polysaccharide chain. A number in combination with an ‘S’ attached to a sugar represents the position of sulfate groups. A number in combination with an ‘OMe’ attached to a sugar represents the position of methyl ether groups.

The marine porphyran degradation was enabled with the investigation of the first marine β‐porphyranases from the Bacteroidetes *Z. galactanivorans*.^[59]^ These enzymes belong to family GH16 and were shown to cleave the β‐1,4‐linkage between β‐d‐galactose and α‐l‐galactose‐6‐sulfate in purified polymeric porphyran resulting in the disaccharide Gal6S‐Gal as the final degradation product.^[59]^ Later a new β‐porphyranase from *Bacteroides plebeius* from family GH86 was identified.^[60]^ Similar studies indicated the importance of GH16 enzymes in the degradation of porphyran by the investigation of further GH16 porphyranases.[^85^, ^89^] Nevertheless, these enzymes are not sufficient on their own for the complete degradation of porphyran (Figure 2). They require a synergistic cleavage of several side group‐removing enzymes which deprotect the polysaccharide chain from functional groups and thereby enable further degradation by the porphyranases. As mentioned above, P450 monooxygenases catalyse demethylation of 6‐*O*‐methyl‐d‐galactose – a monosaccharide that replaces d‐galactose in porphyran in a random manner.[^77^, ^90^, ^91^] Hence, these P450s are crucial for the complete decomposition of porphyran. Besides methylated sugars, porphyran is known to contain l‐galactose‐6‐sulfate.^[92]^ There are reports about putative sulfatase genes in PUL structures presumably targeting porphyran,^[60]^ but they have not been biochemically characterized and their function is not yet confirmed.

### 3.3 Laminarin

Laminarin is one of the most abundant marine polysaccharides.^[27]^ It occurs in brown algae and especially in diatoms (Table 1).^[93]^ It is a highly water soluble glucan which is composed of linear β‐1,3‐linked d‐glucose with β‐1,6‐linked d‐glucose side chains (Scheme 1).^[93]^

Several CAZymes are required for the depolymerisation of laminarin (Figure 2). Laminarinases, the main laminarin‐degrading enzymes, are classified into *endo*‐β‐1,3‐glucanases (laminarinases) (EC 3.2.1.6 and EC 3.2.1.39) and *exo*‐β‐1,3‐glucanases (EC 3.2.1.58). *Endo*‐β‐1,3‐glucanases hydrolyse the β‐1,3 backbone while *exo*‐β‐1,3‐glucanases cleave off glucose from the non‐reducing ends of laminarin oligosaccharides. *Endo*‐acting laminarinases are mainly grouped into families GH16, GH17, GH55, GH64, and GH81, while the GH3 family contains *exo*‐acting laminarinases.^[54]^

While GH16 laminarinases can cleave β‐1,3‐ and β‐1,4‐linkages, GH17 enzymes are highly specific for undecorated β‐1,3 glucans.^[69]^ Two GH16 and GH17 enzymes from *F. agariphila* KMM 3901^T^ and a GH30 enzyme from *Formosa* sp. Hel1_33_131 were investigated as well.^[54]^ The GH16 enzyme had 44 % and 43 % identity with two *endo‐*acting laminarinases from *Z. galactanivorans*. GH17 enzymes are *endo*‐type enzymes specific for β‐1,3‐glucans, while the GH30 family contains enzymes, which are specific for β‐1,6‐glucans. Thereby, *endo*‐acting enzymes from family GH17 are required for the depolymerisation of the laminarin backbone, while *exo‐*acting GH30 enzymes hydrolyse the side chains. This combination of GH17 and GH30 enzymes is necessary for an efficient laminarin depolymerisation.^[54]^ Beside this, there has been a report about a promiscuous GH3‐like laminarinase from *Vibrio campbellii*, which is able to cleave β‐1,3‐linkages as well as β‐1,4‐ and β‐1,6‐linkages (Figure 2).^[55]^ These laminarin‐degrading enzymes are conserved in marine Bacteroidetes. Thus, it was demonstrated that enzymes encoded in both chromosomes of *P. carrageenovora* showed activity on β‐1,3‐glucans. They contain genes for several GH16 *endo*‐1,3‐β‐glucanases. One of the respective PUL structures is conserved in 47 of 52 analysed *Pseudoalteromonas* genomes.^[94]^ Genome analyses of 53 marine bacterial isolates revealed 400 PULs from which 46 PULs (ca. 12 %) are putatively laminarin‐targeting.^[69]^ Thus, the laminarin decomposition plays an important role in the marine polysaccharide turnover.

### 3.4 Ulvan

Ulvan is the major cell wall polysaccharide of macroalgae from the genus *Ulva*.^[62]^ It is a branched, highly sulfated polysaccharide composed of repeating disaccharide units of β‐1,4‐linked d‐glucuronic acid (GlcA) or α‐l‐iduronic acid (IdoA) to α‐1,4‐linked l‐rhamnose‐3‐sulfate (Rha3S). The GlcA can be replaced by β‐1,4‐linked d‐xylose (Xyl) or d‐xylose‐2‐sulfate (Xyl2S) (Scheme 1, Table 1). Also, GlcA side chains at position 2 of Rha3 S have been reported.^[62]^

**Figure 3:**
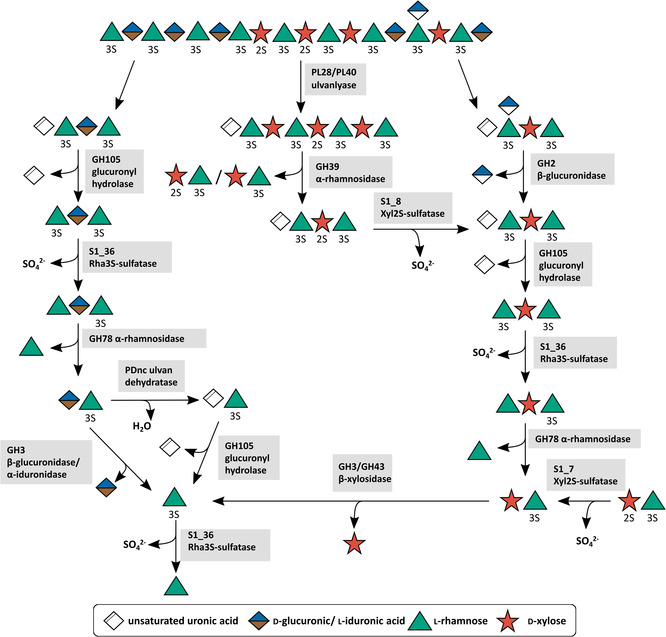
Metabolic ulvan degradation pathway by CAZymes based on current knowledge.[^62^, ^111^] The oligosaccharide on the top represents a section of a larger polysaccharide chain. A number in combination with an ‘S’ attached to a sugar represents the position of sulfate groups. ‘Unsaturated uronic acid’ represents 4‐deoxy‐α‐l‐*threo*‐hex‐4‐enopyranuronic acid.

The first enzymatic decomposition of ulvan by a marine bacterium was reported more than twenty years ago when the first ulvan lyase (EC 4.2.2.–) was discovered.^[95]^ Several other ulvan lyases from the families PL24, PL25, PL28 and PL40 were described in various Bacteroidetes and Proteobacteria.[^61^, ^62^, ^87^, ^96^, ^97^, ^98^, ^99^, ^100^, ^101^, ^102^, ^103^, ^104^, ^105^] They catalyse the initial cleavage step for the degradation of ulvan via an elimination mechanism and cleave the α‐1,4‐linkage between rhamnose‐3‐sulfate and glucuronic or iduronic acid under the formation of an unsaturated uronic acid residue (Δ) at the non‐reducing end. This residue can then be hydrolytically removed by glucuronyl hydrolases of the family GH88 or GH105 (EC 3.2.1.–),[^62^, ^106^, ^107^, ^108^, ^109^] forming 5‐dehydro‐4‐deoxy‐d‐glucuronate. Enzyme functions for the ulvan degradation system of *F. agariphila* KMM 3901^T^ were first predicted by similarity with the help of artificial chromogenic substrates^[107]^ after which the first complete metabolic ulvan degradation pathway was elucidated (Figure 3).^[62]^

In *F. agariphila* the initial depolymerization step is catalysed by ulvan lyases of family PL28 and PL40. The PL28 family ulvan lyase exhibits a type IX secretion signal and an additional ulvan binding module, which facilitates the recognition and binding of polymeric ulvan. As these properties are missing in the PL40 family lyase, it is suggested that it is more likely a membrane‐associated or periplasmatic enzyme. This indicates that its main function is to degrade larger oligosaccharides produced by PL28 family lyase, although it exhibits the same activity against polymeric ulvan.^[62]^ This strategy most probably avoids smaller substrate molecules diffusing away from the bacterial cell. Instead, they are cleaved immediately before or after their uptake into the periplasm by TonB‐dependent transporters (TBDTs). This strategy is also known as the ′selfish′ uptake mechanism.^[110]^ Larger oligosaccharides that are resistant to ulvan lyases, usually contain larger amounts of xylose, as it was shown for xylose‐rich ulvan. A novel *endo‐*rhamnosidase of family GH39 was demonstrated to degrade them. Up to this point, family GH39 was not described to contain rhamnosidases. A BlastP search revealed that this enzyme shows a rather low identity with all other GH39 enzymes.^[107]^ Thus, it was described to be a new type of a GH39 enzyme with a novel activity and most presumably different structural motifs due to the confirmed *endo‐*activity.

Beside these xylose‐containing oligosaccharides, uronic acid‐containing oligosaccharides were described to be resistant to further ulvan lyase degradation as well.^[62]^ At higher ulvan concentration the lyases are inhibited by their own products.^[95]^ Furthermore, the small xylose‐containing oligosaccharides, are resistant to further degradation by lyases or glycoside hydrolases. For a complete depolymerization, a removal of any side chains and protective groups from the particular polysaccharide is necessary. The cleavage of sulfate ester bonds requires a set of specialized sulfatases. On ulvan fragments, sulfatases from the families S1_7, S1_8 and S1_25 showed activity.[^62^, ^111^] An endolytic S1_8 family xylose sulfatase was described to desulfate small xylose‐containing tri‐ and tetrasaccharides like Δ‐Rha3S‐Xyl2S‐Rha3S and Rha3S‐Xyl‐Rha3S. In contrast, exolytic rhamnose‐ and xylose‐ sulfatases from family S1_25 and S1_7 are responsible for desulfation of non‐reducing end rhamnose or xylose residues. This enables a further degradation by several other CAZymes. *Exo‐*rhamnosidases from family GH78 cleave off the non‐reducing end rhamnose residue. In the case of uronic acid‐containing fragments, the responsible GH78 rhamnosidase was shown to be a multimodular CAZyme also containing a family S1_25 sulfatase responsible for desulfating the substrate non‐reducing end rhamnose residue.^[111]^

This was the first characterized multimodular CAZyme involved in ulvan degradation. Multimodular enzymes were revealed before in other Bacteroidetes even the same combination of GH78 and sulfatase was reported for *N. ulvanivorans*,^[26]^ which underlines the importance of this combination of enzyme activities for the utilization of ulvan. The last step of the degradation of ulvan to monomeric sugars is cleavage of the disaccharides Xyl‐Rha3S and GlcA/IdoA‐Rha3S. β‐Xylosidases of family GH3 and GH43 were shown to hydrolyse Xyl‐Rha3S. Two options for the cleavage of GlcA/IdoA‐Rha3S were recently discovered by us.^[111]^ A β‐glucuronidase/α‐iduronase from family GH3 or a novel polysaccharide dehydratase in combination with unsaturated glucuronyl hydrolase from family 105 were able to cleave the uronic acid‐containing disaccharides (unpublished).

## 4 Saccharification Processes for Marine Sugars

The elucidation of these marine polysaccharide utilization systems enables the use of algal biomass for fermentation processes as well as the production of biofuels and high value fine chemicals. Currently, algal biomass is treated as waste and accumulates in very large amounts due to the high growth rate of algae,^[112]^ making it a cheap and easily accessible source of raw materials. For their use in biotechnological applications, biorefinery concepts were published mostly for the efficient saccharification and fermentation of brown algae carbohydrates. Among these, the metabolic engineering of *Saccharomyces cerevisiae* for the fermentation of mannitol and alginate degradation products to ethanol was reported.^[113]^ The metabolic engineering of *Escherichia coli* led to the creation of a strain that is able to degrade, take up and metabolize alginate under the production of bioethanol.^[114]^ The clarification of the metabolic pathway for 3,6‐anhydro‐l‐galactose enabled the use of the red algae polysaccharides agar and carrageenan for the same purpose.^[115]^ The direct bioconversion of brown algae into ethanol was reported for *Defluviitalea phaphyphila*.^[116]^ Another milestone in the biofuel production from algae was the engineering of the yeast *S. cerevisiae* for enzymatic hydrolysis of laminarin from brown macroalgae for the production of bioethanol.^[117]^ Recent reviews summarize biofuel feedstocks including macroalgal^[118]^ and related genetic engineering approaches.^[119]^

The production of *meso‐*2,3‐butanediol from glucose was demonstrated by metabolic engineering of *Bacillus licheniformis*.^[120]^ Glucose can be produced using the widespread glucanases of various organisms to degrade the green algal glucans and the brown algal laminarins. This enables the 2,3‐butanediol fermentation using marine polysaccharides as feedstock. Besides ethanol, hydrogen is a promising energy carrier. The fermentative hydrogen evolution was reviewed showing biochemical pathways for the production of hydrogen by various microorganisms.^[121]^ The reported hydrogen generation systems involving bacteria growing on first or second generation plant sources^[122]^ can easily be adapted to the use of algal biomass as the investigation of fermentative pathways starts with monosaccharides that can be provided by either land plants or algae. Thus, the hydrogen evolution using the hyperthermophilic bacteria *Thermotoga neapolitana* on biomass of the green alga *Chlamydomonas reinhartii* was reported.^[123]^ The production of hydrogen from xylose is also possible using an *in vitro* enzyme cascade,^[124]^ showing that hydrogen evolution can also work cell‐free. To increase the yield of produced biofuels from macroalgae several pretreating methods were described recently.[^125^, ^126^]

In principle, the application of carbohydrates in biotechnological processes for fermentation requires the possibility to fully degrade the respective carbohydrate to the monomeric level and the ability to metabolize the corresponding monosaccharides released by the polysaccharide degradation. Thus, the more complex the polysaccharide is, the more CAZymes are required for its degradation. If the polymer contains rare sugars, there are often only a few microorganisms which are able to metabolize them. For the production of ethanol, yeasts like *S. cerevisiae* are often used because they exhibit a high ethanol tolerance.^[127]^ Even if yeasts have been implemented in the production from bioethanol from brown algae^[117]^ they still often lack genes in the metabolic pathways encoding for proteins for the conversion of pentose sugars like xylose and arabinose.^[128]^ Enabling a usage of these sugars would require metabolic engineering,^[129]^ complicating the use of algal biomass in yeast fermentation processes. However, especially rare sugars can not only serve as carbon source for fermentation, but also be a value product on their own. Thus, 3,6‐anhydro‐l‐galactose can be isolated from red algae and it was reported to exhibit skin whitening and anti‐inflammatory properties.^[130]^

## 5 Conclusion

In contrast to their terrestrial counterpart, the metabolic degradation of marine polysaccharides is currently still underexplored. Marine microorganisms provide enzymatic toolboxes for the successive degradation of these carbohydrates into monomeric sugars. The acquired insight of the metabolic polysaccharide utilization greatly expands the possibility to use algal waste for recycling in biorefinery processes to high value materials with even beneficial effect for the environment. The research on this topic is still in its infancy regarding the huge diversity of marine polysaccharides and still much scientific work is necessary in this field to overcome the bottlenecks for producing fermentable saccharide fragments from algae for the production of valuable chemicals.

## Conflict of interest

The authors declare no conflict of interest.

## Biographical Information

*Marcus Bäumgen studied biochemistry at Greifswald University where he received his PhD in 2020 in the group of Prof. Bornscheuer. After focusing on research with Baeyer‐Villiger monooxygenases, he switched his interest to the marine polysaccharide utilization and functional characterization of marine carbohydrate‐active enzymes within the DFG‐funded research unit FOR2406 “Proteogenomics of Marine Polysaccharide Utilization” (POMPU)*.

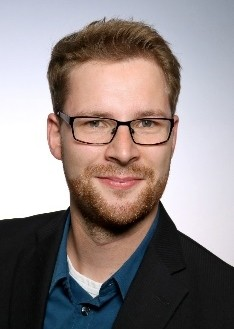

## Biographical Information

*Theresa Dutschei studied biochemistry at Greifswald University and received her M.Sc. degree in the group of Prof. Bornscheuer in the field of applied marine carbohydrate saccharification and utilization. Since 2019 she also works in the DFG‐funded POMPU‐project on the functional characterization of marine carbohydrate‐active enzymes*.

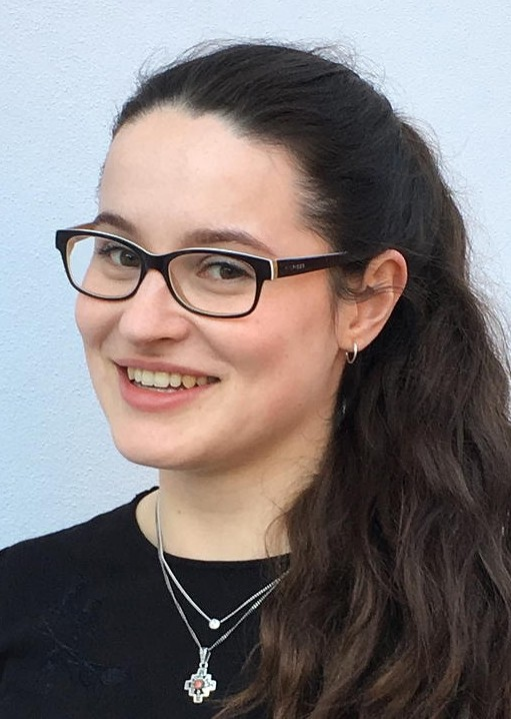

## Biographical Information

*Uwe T. Bornscheuer studied chemistry and received his PhD in 1993 at Hannover University followed by a postdoc at Nagoya University (Japan). In 1998, he completed his Habilitation at Stuttgart University about the use of lipases and esterases in organic synthesis. He has been Professor at the Institute of Biochemistry at Greifswald University since 1999. Beside other awards, he received in 2008 the BioCat2008 Award. He was just recognized as ‘Chemistry Europe Fellow’. His current research interest focuses on the discovery and engineering of enzymes from various classes for applications in organic synthesis, lipid modification, degradation of plastics or complex marine polysaccharides*.